# Food Consumption Trends in Japanese Children and Adolescents: The National Health and Nutrition Survey, 2001–2019

**DOI:** 10.3390/foods14081392

**Published:** 2025-04-17

**Authors:** Chisa Shinsugi, Hidemi Takimoto

**Affiliations:** 1 National Institute of Health and Nutrition, National Institutes of Biomedical Innovation, Health and Nutrition, Osaka 566-0002, Japan; 2 Department of Empirical Social Security Research, National Institute of Population and Social Security Research, Tokyo 100-0011, Japan

**Keywords:** food consumption, National Health and Nutrition Survey, children, adolescents, Japan

## Abstract

Background: While unhealthy dietary trends, such as elevated confectionery consumption and decreased fruit intake, have been documented in adults, the longitudinal patterns of food consumption during childhood remain inadequately characterized. This study aimed to describe national trends in food group intakes among children and adolescents in Japan. Methods: Data on participants aged 1–19 years in the National Health and Nutrition Survey from 2001 to 2019 (*n* = 37,072) were used in this study. A 1-day, semi-weighed, household-based dietary record was used to assess dietary intake. Results: Decreasing trends in the intakes of animal-based foods, potatoes and starches, sugars and sweeteners, fruits (annual percentage change [APC] range: −5.04 to −0.62), algae, fish and shellfish (APC range: −3.22 to −2.02), eggs, milks, fats and oils, and confectionery were observed, while intakes of meats (APC range: 1.02 to 1.92) and beverages (APC range: 1.36 to 2.51) increased. Consumption of plant-based foods, cereals, legumes, nuts and seeds, vegetables, and mushrooms was mostly unchanged, whereas variable intakes of seasonings and spices were observed. Conclusions: This study showed that the intakes of many food groups (e.g., fruits, fish and shellfish, and milk) decreased among children and adolescents, but some increased (e.g., meats and beverages) or remained stable (e.g., cereals and vegetables). Further evidence is needed to enable policymakers to set target interventions to improve children’s diets.

## 1. Introduction

Adequate diet quality and quantity from early childhood are essential for healthy growth and development. In recent years, unhealthy dietary trends have been observed in Japanese adults. Vegetable and fruit intakes among adults have decreased in many regions [1]. Among young women, consumption of fish, shellfish, and seaweed has been declining, while intakes of meat and soft drinks have increased [2]; higher confectionery consumption among those with obesity and decreased intakes of fruit and dairy products among the non-obese have also been reported [2]. Unhealthy dietary habits have also been identified among children and adolescents. A study [3] on preschool children demonstrated that lower parental interest in nutritional balance was associated with reduced food diversity. Moreover, a study [4] of children and adolescents aged 3 to 17 years found that high consumption of highly processed foods, such as white rice, noodles, and confectioneries, was linked to lower diet quality. However, these studies capture dietary habits at a single point in time, and how the dietary habits of younger populations are changing remains unclear. In Japan, under the School Lunch Act, school lunches are served with the same menu for all children in each compulsory elementary and junior high school and provide more than one-third of the daily nutritional requirements for each age group from 6 to 14 years, as specified in the Dietary Reference Intakes for Japanese (DRIs), except for special cases such as children with food allergies [5,6]. School lunch (a full meal) consists of a staple food (e.g., cooked rice, bread, or noodles), a main dish, side dishes, and milk [6]. School lunch programs are implemented in almost all elementary schools (about 99%) and over 91% of junior high schools. They are served approximately 190 times a year. A previous study reported that the overall diet quality on non-school days was worse than on days with school lunches [7]. The quality of diet for adolescents who are not eligible for school lunches is also a concern. Furthermore, the Japanese Food Guide Spinning Top, developed by the Ministry of Health, Labour and Welfare and the Ministry of Agriculture, Forestry and Fisheries, is a food guide that shows what and how much food to eat in a day to promote a healthy diet [8]. This is a national standard for people aged 6 years and older, but it does not exist for younger children.

Food consumption may change over time due to social factors [9], including food affordability in school lunches and at the household level, food availability in local grocery stores, commercial marketing (e.g., attractive confectionery and beverage food labels, social media promotions of fast food), and relevant policies and regulations. Identifying long-term trends in children’s food consumption can help identify challenges within the food environment and indicate the necessity of public health interventions. Furthermore, as unhealthy dietary habits in childhood can lead to diabetes and chronic diseases in adulthood [10], monitoring eating habits may help to prevent long-term adverse health outcomes.

In Japan, nutrition education in schools is carried out during school lunchtime and home economics classes. Provision of daily school lunches using locally available foods and seasonal ingredients is regarded as a useful tool to teach healthy eating habits, while monthly menu lists enable communication with children and their families to promote healthy food choices. Dietitians, therefore, play an important role as health promoters within the community by providing nutrition counselling and planning school lunch menus. By providing nutrition information in public preschools and primary schools, they can alert children and parents to nutrient excesses or deficiencies based on the latest food consumption trends. Moreover, it is crucial for policymakers and public health experts to understand these current food consumption patterns to develop food-based dietary goals for young children under 6 years, and to evaluate individual food groups as sources of essential nutrients and review the DRIs, which is revised every five years. To support these varied interventions to improve children’s diets, we aimed to describe national trends in food consumption among Japanese children and adolescents from 2001 to 2019.

## 2. Materials and Methods

### 2.1. Study Design

This study utilized data from the National Nutrition Survey (NNS, 2001–2002) and the National Health and Nutrition Survey (NHNS, 2003–2019) conducted by the Ministry of Health, Labour and Welfare in Japan [11] in accordance with the Health Promotion Act. Participants were household members aged 1 year and over living in 300 census enumeration areas selected using a stratified cluster sampling across all 47 prefectures in Japan. However, there were some exceptions. The 2004 survey included 298 census enumeration areas due to the Mid-Niigata Prefecture Earthquake. The 2012 and 2016 surveys were carried out in 475 and 462 census enumeration areas, respectively. The method of cluster sampling in 2012 and 2016 differed from that in the other years, and weighting was performed to correct for the resulting differences between the numbers of households in each prefecture. Three prefectures (Iwate, Miyagi, and Fukushima) were excluded from the 2011 survey because of the Great East Japan Earthquake, and one prefecture (Kumamoto) was excluded from the 2016 survey owing to the Kumamoto Earthquake. Household response rates for each year ranged from 44.4% in 2016 to 83.2% in 2003 [11]. The detailed methodology of the NNS and NHNS has been published previously [12,13,14]. Ethical review and approval were waived for this study because only anonymized data were used. Of the 2001–2019 survey participants, 37,072 (18,939 boys and 18,133 girls) aged 1–19 years were included in this analysis. Due to the lack of recommended amounts in the Japanese Food Guide Spinning Top for younger age groups and considering the elementary school entry age (April of the year following their sixth birthday) and school lunch eligibility, food consumption patterns were assessed in three age categories: young children (1–6 years), school-age children (7–14 years), and adolescents (15–19 years).

### 2.2. Dietary Assessment

Dietary data were collected using a 1-day, semi-weighed, household-based dietary record on a usual day that was neither a Sunday nor a public holiday. Household meal preparers recorded the names of food ingredients and beverages, weight, and leftover amounts of food for each child in the dietary record. To account for shared dishes within the household, the approximate proportions of each food were assigned to individual household members to estimate individual food intake. The amount eaten at school lunch was also recorded based on school lunch recipes collected from relevant authorities. When food weight was missing, trained dietitians converted the portion sizes or amounts of foods according to the food number lists using an official food item booklet that contains standard portions of frequently consumed dishes. During the survey, trained dietitians visited each household at least once a day to check the dietary record, including any leftover portions from school lunches. The NNS and NHNS have used the same dietary assessment methods since the introduction of individual nutrient and food group intake assessments in 1995.

The individual dietary record data consisted of dishes and their food items, the cooking methods (e.g., boiling or roasting), and the amount of each food item consumed. Each food item was assigned a unique food number according to the Standard Tables of Food Composition in Japan (5th edition for the 2001–2004 survey; 5th revised and enlarged edition for the 2005–2010 survey; 2010 edition for the 2011–2017 survey; 2015 edition for the 2018–2019 survey) [15,16,17,18]. The energy and nutrient contents of each dish consumed are calculated based on the Standard Tables of Food Composition. The following food groups were included in this study: animal-based foods, plant-based foods, cereals, potatoes and starches, sugars and sweeteners, legumes, nuts and seeds, vegetables, fruits, mushrooms, algae, fish and shellfish, meats, eggs, milk, fats and oils, confectionery, beverages, seasonings and spices. Animal-based foods comprise fish and shellfish, meats, eggs, milk, butter, and animal fats and oils. Plant-based foods consist of cereals, potatoes and starches, sugars and sweeteners, legumes, nuts and seeds, vegetables, fruits, mushrooms, algae, margarine, vegetable fats and oils, other fats and oils, confectionery, beverages, seasonings and spices [19]. Cereals are further divided into two categories: rice and rice products and wheat flour and wheat products. The changes in food group classifications in NNS and NHNS have been described in detail elsewhere [14].

### 2.3. Statistical Analysis

The mean and standard error (SE) were calculated by sex, age group, and survey year. Trend analyses were performed using Stata Version 16.1 (StataCorp, College Station, TX, USA) and the Joinpoint Regression Program (Joinpoint Regression software, version 5.0.2; National Cancer Institute, Rockville, MD, USA). Joinpoint regression analysis uses statistical criteria to determine the minimum number of linear segments required to describe a trend and perform the annual percentage change (APC) for each segment. The Monte Carlo Permutation method was used to test whether a change in trend was statistically significant. The Weighted Bayesian Information Criterion was employed for the model selection to account for effect sizes [20]. Differences were considered statistically significant at *p* < 0.05.

## 3. Results

**Figure 1:**
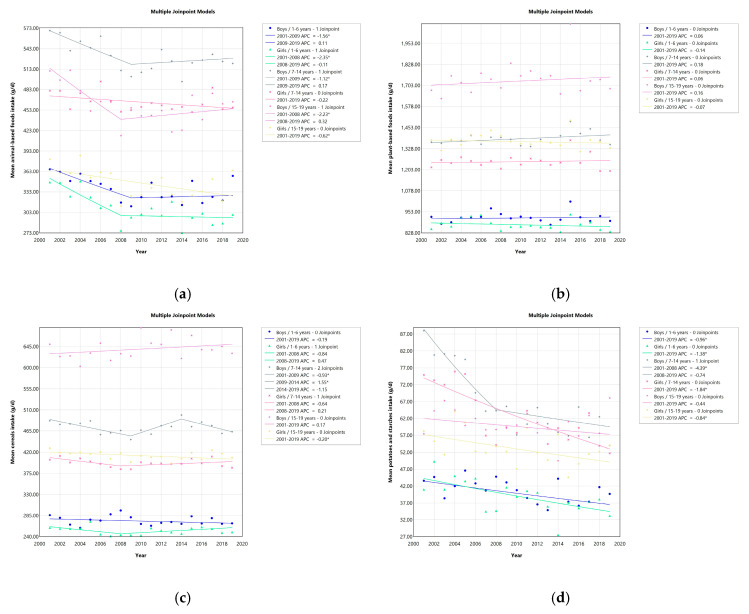

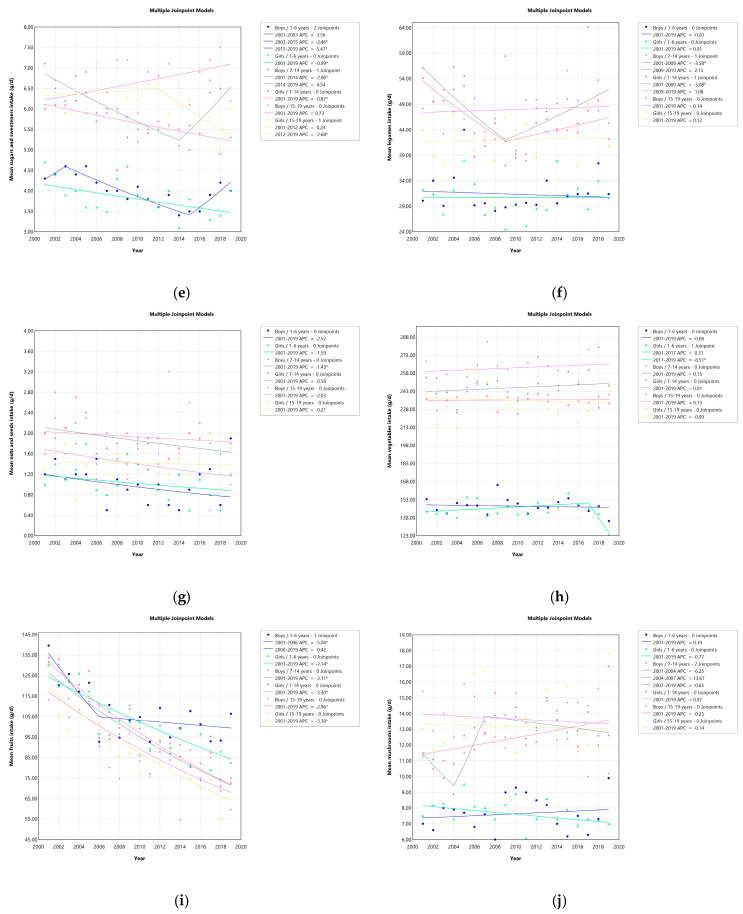

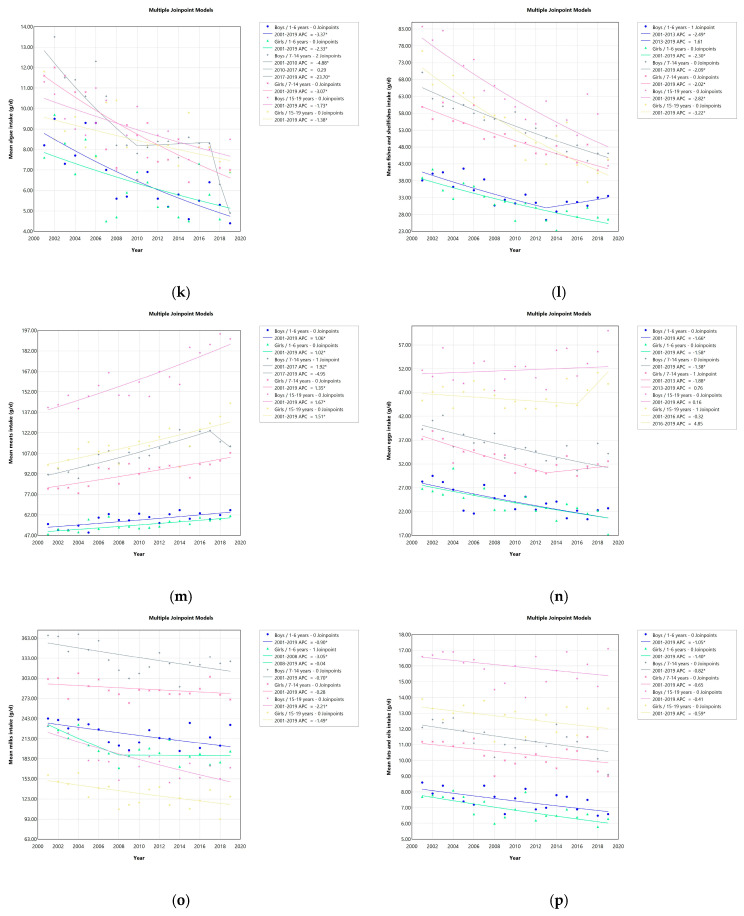

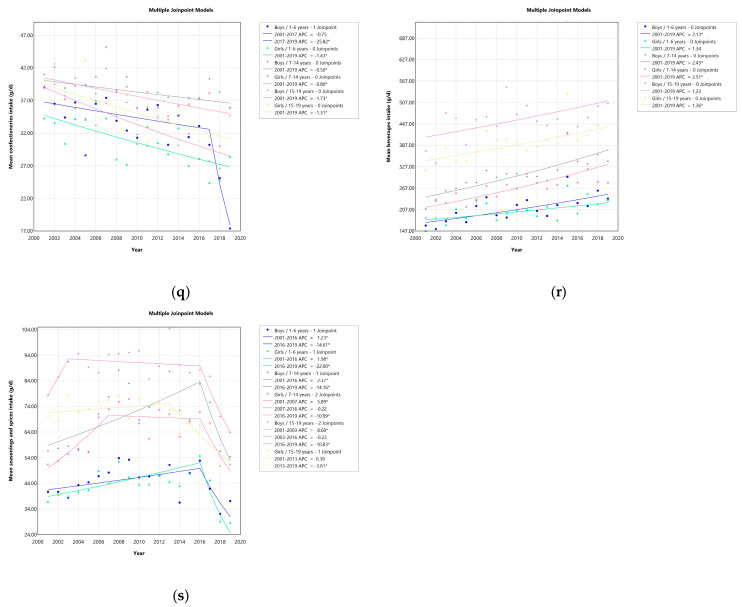
Trends in food consumption by sex and age from 2001 to 2019; annual percentage change (APC). * *p* < 0.05. Animal-based foods refer to fish and shellfish, meats, eggs, milk, butter, and animal fats and oils, while plant-based foods include cereals, potatoes and starches, sugars and sweeteners, legumes, nuts and seeds, vegetables, fruits, mushrooms, algae, margarine, vegetable fats and oils, other fats and oils, confectionery, beverages, seasonings and spices: (**a**) animal-based foods intake, (**b**) plant-based foods intake, (**c**) cereals intake, (**d**) potatoes and starches intake, (**e**) sugars and sweeteners intake, (**f**) legumes intake, (**g**) nuts and seeds intake, (**h**) vegetables intake, (**i**) fruits intake, (**j**) mushrooms intake, (**k**) algae intake, (**l**) fishes and shellfishes intake, (**m**) meats intake, (**n**) eggs intake, (**o**) milks intake, (**p**) fats and oils intake, (**q**) confectioneries intake, (**r**) beverages intake, and (**s**) seasonings and spices intake.

Trends in food consumption by sex and age are presented in Figure 1 and Supplementary Tables S1–S6. Decreasing trends in the intakes of animal-based foods, potatoes and starches, sugars and sweeteners, fruits, algae, fish and shellfish, eggs, milks, fats and oils, and confectionery were reported, while intakes of meats and beverages increased. Consumption of plant-based foods, cereals, legumes, nuts and seeds, vegetables, and mushrooms was mostly unchanged, whereas variable intakes of seasonings and spices were observed. The number of survey participants decreased over the years. Supplementary Table S7 shows APCs in food consumption by sex and age.

While declining trends in animal-based food intake were observed in all age groups up to 2008/2009 (APC range: −2.35 to −1.12)—with the exception of school girls—intake of plant-based foods in all age categories was unchanged. Cereal intake remained unchanged except for school boys (APC −0.93 [2001–2009] and 1.55 [2009–2014]), with the average intake in 2019 ranging from 249.5 g per day for young girls to 630.5 g per day for adolescent boys. While no changes were observed in the consumption of rice and rice products, decreases in the consumption of wheat flour and wheat products were observed in girls of all ages and in school boys. Boys in this age group consumed an average of 38.5 g less wheat per day in 2019 (85.7 g) compared to 2001 (124.2 g).

Declining trends in the intake of potatoes and starches were observed in some age categories (APC −1.38 for young girls; APC −4.39 [2001–2008] for school boys; APC −1.84 for school girls). For example, school boys ate an average of 34.0 g fewer potatoes and starches per day in 2019 (54.0 g) compared to 2001 (88.0 g). Slight reductions in the consumption of sugars and sweeteners were observed in some age groups (APC −2.46 [2003–2015] for young boys; APC −2.06 [2001–2014] for school boys; APC −2.68 [2012–2019] for adolescent girls). Young boys consumed an average of 0.3 g less sugars and sweeteners per day in 2019 (4.0 g) compared to 2001 (4.3 g).

Vegetable intake remained unchanged—except for young girls (APC −8.51 [2017–2019])—with an average intake in 2019 of approximately 130 g per day for young children and approximately 240 g per day for school children and adolescents. Decreasing trends in fruit consumption were observed in all age groups (APC range: −5.04 to −2.14). For example, among adolescent boys, fruit consumption decreased by an average of 53.6 g per day (from 113.2 g per day in 2001 to 59.6 g in 2019).

Average daily intake of fish and shellfish declined in all age groups (APC range: −3.22 to −2.02). Average consumption decreased by 41.3 g per day among adolescent boys (from 83.7 g in 2001 to 42.4 g per day in 2019). Increasing trends in meat intake were observed in all age groups (APC range: 1.02 to 1.92). For example, average daily meat consumption increased by 50.3 g per day among adolescent boys (from 140.5 g per day in 2001 to 190.8 g per day in 2019). Declining intake of eggs was observed in young and school-aged children (APC range: −1.88 to −1.38)—including a 9.6 g per day decrease in average egg intake among young girls (from 26.8 g per day in 2001 to 17.2 g in 2019). However, an increasing trend in egg consumption was observed in adolescent girls (APC 4.85 [2016–2019]). Decreasing milk intake was observed in some age groups (APC −3.05 [2001–2008] for young girls; APC −2.21 for adolescent boys; APC −1.49 for adolescent girls). Milk intake decreased by an average of 38.7 g per day among school boys (from 367.0 g in 2001 to 328.3 g per day in 2019).

Confectionery consumption declined in all age groups (APC range: −25.82 to −0.50). For example, average daily confectionery consumption decreased by 21.6 g among young boys (from 39.0 g per day in 2001 to 17.4 g in 2019). Beverage intake increased in all age groups (APC range: 1.36 to 2.51). School boys consumed an average of 133.4 g more beverages per day (from 209.3 g per day in 2001 to 342.7 g in 2019).

## 4. Discussion

This study analyzed national trends in food consumption among Japanese children and adolescents over 19 years. While decreasing trends in the intakes of about half of the food groups were observed, there were increasing trends in the intakes of meats and beverages. Consumption of about one-third of the food groups—with a few exceptions—was unchanged, whereas both increased and decreased intakes of seasonings and spices were observed over time across age groups.

This study showed a decreasing intake of animal-based foods over time, while plant-based food intake remained unchanged. To promote sustainable and healthy diets, the FAO/WHO recommends reducing the consumption of animal-source products due to their high environmental impact and poorer health outcomes [21]. A previous study [22] in young people aged 18–20 years reported that a healthy dietary pattern characterized by high intake of vegetables, seaweeds, soy products, and fruit was related to a lower risk of obesity (BMI of 25 or over). Another study [23] observed that higher intakes of potatoes, pulses, vegetables, fruits, and dietary fiber were linked to a lower prevalence of constipation, which is a common problem in preschool children. It is crucial to accumulate evidence on the health impacts of sustainable and healthy diets that are friendly to Japanese food culture. Moreover, consistent with the present study, a study [24] in the US reported that the intakes of legumes, nuts, and seeds among adolescents remained unchanged over time, although the intake of legumes among young and school-aged children had increased. Efforts should be made to further accelerate the shift towards recommended proteins such as legumes, nuts, and seeds by improving accessibility and increasing food choice (for example, by promoting the development of new legume-based products such as soya noodles). Regularly reviewing the dietary habits to monitor the consumption of these nutrient-rich foods and providing parents and children with information on these healthy food choices through familiar channels, such as nursery or school letters, would be important.

While no changes were observed in the intakes of cereals (except for school boys) and rice and rice products, the intake of wheat flour and wheat products decreased in girls of all ages and school boys. This may be partially because of the influence of the Washoku School Lunch Project [25,26] promoting Japanese cuisine in school meals—an initiative by the Ministry of Agriculture, Forestry and Fisheries to preserve the traditional Japanese diet after it was designated as a UNESCO Intangible Cultural Heritage in 2013. Although a previous study [27] in US youth reported a modest increase in whole-grain intake among high-income groups, it would be crucial to foster an environment that enhances access to cereals with better health benefits, such as promoting brown rice in school lunches, regardless of socio-economic status.

Vegetable intake was observed to remain unchanged in this study, with an average intake in 2019 of approximately 130 g per day for young children and approximately 240 g per day for school children and adolescents. The third phase of Healthy Japan 21—a 12-year (2024–2035) comprehensive national health and nutrition policy based on the Health Promotion Act—encourages Japanese people to increase vegetable intake to at least 350 g per day [28]. Moreover, WHO recommends consuming more than 400 g of fruits and vegetables per day to improve overall health and reduce the risk of noncommunicable diseases [10]. Although these suggestions are not directly applicable to children, a previous study found that children and adolescents from lower-income households were less likely to eat vegetable dishes [29]. Recent rises in the prices of vegetables and other foodstuffs may have caused changes to household purchases of fresh food that were not captured in this analysis, and therefore, it is necessary to continue monitoring future trends in child vegetable intake.

Inconsistent with a previous study in Western European teenagers [30], the fruit intake declined drastically over the study period. For example, among adolescent boys, fruit consumption decreased by an average of 53.6 g per day (from 113.2 g per day in 2001 to 59.6 g in 2019). The Japanese Food Guide Spinning Top recommends that children aged 6 years and older should eat two servings (approximately 200 g by weight) of fruit per day [8]. Vitamin C intake has decreased [31], particularly among adolescents, who consume levels below the estimated average requirement (EAR) of 85 mg per day (75 mg per day for boys and 81 mg per day for girls in 2019). Furthermore, growing socioeconomic inequalities in fruit consumption have been reported in some Western European countries [30]. Urgent action is therefore needed to increase fruit intake among children and adolescents, such as encouraging parents to increase the availability of fruit at home and supporting the provision of fruit at low prices in school cafeterias.

Substantial reductions in the intake of fish and shellfish were found in this study. This downward trend is of concern because it has been reported that lower intakes of fish, eicosapentaenoic acid (EPA), and docosahexaenoic acid (DHA) are related to higher prevalence of depressive symptoms in boys aged 12–15 years [32]. Moreover, school children aged 10–11 years from lower-income households have been reported to consume less fish and shellfish, especially on days without school lunch [33], suggesting that interventions should be targeted to high-risk groups. Due to the geographical nature of Japan as an island nation, fish and shellfish are readily available everywhere in the country, and the tradition of eating raw fish, such as sushi, is ingrained from childhood. However, the increase in dual-income households and the demands of caregiving for older people, which constrain the time parents can allocate to cooking and eating, along with the proliferation of simplified diets and the difficulty children face in removing fish bones, may be contributing to a decline in fish consumption. Fish is rich in polyunsaturated fatty acids, and urgent action is required to ensure healthy fat choices from an early age.

By contrast, the present study showed significant increases in meat intake, with the meat intake in 2018 ranging from 59.1 g per day for young girls to 194.4 g per day for adolescent boys. Compared to the global mean consumption of meat (unprocessed red meat and processed meat) for children and adolescents (birth to 19 years) in 2018 (58 g per day) [34], the meat intake of Japanese children and adolescents was considerably higher. A previous study [35] showed that meat, dairy products, and confectionery comprised the main dietary sources of saturated fatty acid (SFA) intake in Japanese school children. A meta-analysis [36] found that reducing SFA intake in children reduced total and low-density lipoprotein (LDL) cholesterol levels and diastolic blood pressure without adversely affecting growth and development. Meat is one of the most important sources of protein for the growth and development of children and adolescents. However, the International Agency for Research on Cancer has classified the consumption of processed meat as carcinogenic to humans (Group 1) and red meat as probably carcinogenic to humans (Group 2A) and concluded that each 50 g portion of processed meat eaten daily increases the risk of colorectal cancer by 18% [37]. It is therefore imperative to educate parents and children about the type and the amount of meat they consume (e.g., white meat, lean meat), based on the latest scientific findings.

Changes in egg consumption varied by age group, with declining consumption trends observed among young and school children, whereas increased consumption was found among adolescent girls. Although egg allergy in early childhood requires caution, egg consumption is associated with higher dietary quality and better nutritional status [38]. Furthermore, a meta-analysis in the US found that moderate egg consumption (up to one egg per day) was associated with a reduced risk of cardiovascular disease in Asian populations [39]. As these were studies of adults, further research is needed on age-appropriate egg consumption for children and adolescents.

Declining milk intake was observed in some age groups, and adolescents had lower intakes than other age groups. This may be due to the inclusion of milk in school lunches as stipulated in the Ordinance for Enforcement of the School Lunch Act, which significantly increased the milk intake of school children compared to adolescents who did not receive school lunches. Given that the mean calcium intake of adolescents in 2019 was lower than the recommended dietary allowance (RDA) in the 2020 DRIs [31], policymakers may need to take measures to improve the food environment and to provide information to encourage adolescents to consume more milk and other calcium-rich foods.

Consistent with a previous study in German children and adolescents [40], declining trends in the intake of fats and oils were observed in young and school children. This may be partly explained by a shift from home-prepared foods to packaged foods and/or foods from outside sources, such as convenience stores, or by efforts by food suppliers to reduce the amount of fats and oils in food products. Moreover, the present study found decreases in confectionery intake, which is consistent with a previous study in French children [41]. A previous study [42] reported that almost half of Japanese children and adolescents consumed more than 5% of daily energy in the form of free sugars and found a positive association between free sugar intake and the consumption of sugars and confectionery. WHO suggests that both adults and children should reduce their intake of free sugars to less than 10% of total energy intake (strong recommendation) or even further to below 5% of total energy intake (conditional recommendation) [43]. Therefore, school dietitians should continue to advise children and parents to avoid excessive sugar and sweeteners.

By contrast, increased beverage consumption was observed in the present study, which is inconsistent with the findings from other high-income countries [44,45]. Although the WHO Western Pacific Region has called on Member States to take action to protect children from the harmful impact of food marketing, such as foods high in saturated fats, trans-fatty acids, free sugars, or salt [46], Japan lags behind in creating a healthy food environment for children. For example, there is no policy action on the marketing of foods or non-alcoholic beverages to children. Addressing these gaps is an urgent public health issue.

The intake of seasonings and spices in many age groups increased over the 2000s, but more recent downward trends are desirable from the perspective of salt reduction. Seasonings are the main source of salt intake for Japanese people [47], and the 2019 mean salt equivalent among children and adolescents was above the tentative dietary goal for preventing lifestyle-related diseases (DG) as set by the 2020 DRIs [31]. Nevertheless, emerging concerns have been raised about high salt intake in young adults from processed foods such as instant Chinese noodles and Japanese curry sauce [47].

This study has several limitations. First, although the subjects were randomly selected across the country, the household-level response rate was relatively low. Additionally, the individual-level response rate could not be considered due to unavailability. Second, self-reported dietary assessments are subject to systematic and random errors. Food intake estimated from a 1-day dietary record may not represent long-term habitual intake. Third, the potential for underreporting of energy intake in young children (1–5 years) and adolescents (15–19 years) and in children with obesity has previously been reported [48] and cannot be ruled out in the present study. This study did not include breast milk or formula consumption in children aged 1–2 years. Fourth, declining trends in the consumption of many foods may have been observed because total energy intake decreased, but the data sources did not disclose the values per 1000 kcal. Fifth, although different households are invited to participate in the survey each year, multiple children from a single household may be included in the survey, which may not eliminate internal dependencies in the data. Sixth, children and adolescents with various illnesses requiring specific dietary regimens such as food allergies and diabetes could not be excluded from this study because the survey did not collect this information. Lastly, the consistent seasonal timing of the surveys (mainly in November), which is useful to observe annual trends, may have constrained the observed diversity of food consumption. While these limitations must be considered when interpreting the findings, we presented the annual trends in food consumption using nationally representative data for Japanese children and adolescents over 19 years. Further research on children’s dietary intake is needed, including assessing the adequacy of dietary intake and dietary diversity using international indicators.

## 5. Conclusions

This study found that the intakes of many food groups (e.g., fruits, fish and shellfish, and milk) among Japanese children and adolescents decreased from 2001 to 2019, although some food groups increased (e.g., meats and beverages) or remained stable (e.g., cereals and vegetables). These findings underscore the importance of enhancing literacy on sustainable and healthy food choices among parents and children at every stage of development, from nursery to higher education. Additionally, improving the food environment to ensure these healthy options are affordable and accessible is essential. Continued annual monitoring of food consumption is recommended, and further evidence is needed to enable policymakers to set target interventions to improve children’s diets.

## Data Availability

The data used in this study can be obtained from the survey reports of the Ministry of Health, Labor and Welfare (https://www.mhlw.go.jp/bunya/kenkou/kenkou_eiyou_chousa.html, accessed on 22 February 2024).

## Supplementary Materials

The following supporting information can be downloaded at https://www.mdpi.com/article/10.3390/foods14081392/s1, Table S1. Trends in food consumption in young boys aged 1–6 years from 2001 to 2019. Table S2. Trends in food consumption in young girls aged 1–6 years from 2001 to 2019. Table S3. Trends in food consumption in schoolboys aged 7–14 years from 2001 to 2019. Table S4. Trends in food consumption in schoolgirls aged 7–14 years from 2001 to 2019. Table S5. Trends in food consumption in adolescent boys aged 15–19 years from 2001 to 2019. Table S6. Trends in food consumption in adolescent girls aged 15–19 years from 2001 to 2019. Table S7. Annual percentage change (APC) in food consumption among Japanese children and adolescents by sex and age from 2001 to 2019.
