# Supplementary material for: Food Consumption Trends in Japanese Children and Adolescents: The National Health and Nutrition Survey, 2001–2019
Source: Foods. 2025 Apr 17;14(8):1392. doi: 10.3390/foods14081392 (PMC12027389; doi:10.3390/foods14081392)
Supplement: Supplementary file 1 [file foods-14-01392-s001.zip › foods-3550299-supplementary.pdf]

Supplementary Tables

Table S1. Trends in food consumption in young boys aged 1–6 years from 2001 to 2019.

	2001	2002	2003	2004	2005	2006	2007	2008	2009	2010	2011	2012	2013	2014	2015	2016	2017	2018	2019	*P* for trend
n	396	346	347	263	229	276	243	204	238	236	209	846	197	181	182	611	197	181	105	
Total food weight (g/d)																				
Mean	1,289.2	1,244.7	1,239.9	1,278.4	1,269.5	1,267.9	1,310.9	1,257.2	1,226.4	1,249.8	1263.5	1,227.3	1,202.5	1,220.0	1,362.9	1,237.6	1,224.0	1,248.2	1,255.5	0.105
SE	20.6	22.4	20.2	25.1	24.7	25.0	27.7	24.8	22.6	24.3	26.8	12.6	28.3	26.4	30.8	13.9	26.8	29.0	32.4	
Animal-based foods (g/d)																				
Mean	366.1	362.6	348.9	359.5	348.8	344.8	337.3	317.4	312.0	325.2	346.4	325.2	326.4	313.8	349.1	316.8	325.7	320.3	356.4	0.044
SE	10.3	10.4	10.1	12.3	11.8	10.9	11.5	12.1	11.7	11.7	12.1	6.0	14.0	12.7	13.6	6.9	12.7	14.0	18.4	
Plant-based foods (g/d)																				
Mean	923.1	882.1	891.0	918.9	920.7	923.1	973.5	939.8	914.4	924.6	917.1	902.2	876.1	906.2	1,013.8	920.7	898.3	927.9	899.1	0.979
SE	17.0	18.0	16.9	20.8	21.5	21.7	23.4	20.8	19.4	21.4	23.8	10.6	23.2	23.5	25.6	11.7	23.1	24.5	29.3	
Cereals (g/d)																				
Mean	285.9	280.1	265.6	258.5	276.1	274.1	287.9	296.1	281.2	267.0	263.1	269.4	271.1	267.2	283.4	268.0	279.4	267.3	268.3	0.457
SE	5.5	5.7	5.7	6.0	5.9	7.1	6.8	8.0	7.2	7.1	6.8	3.3	7.0	6.9	7.7	3.4	7.3	7.0	8.7	
Rice and Rice products (g/d)																				
Mean	206.3	208.5	191.4	195.2	201.1	193.8	208.2	216.5	202.9	191.4	192.2	198.3	198.7	196.7	207.4	197.7	209.0	196.5	193.1	0.696
SE	5.0	5.3	5.2	5.7	5.8	5.9	6.3	7.4	7.0	6.7	6.3	3.3	6.9	7.5	7.8	3.6	7.8	7.4	9.8	
Wheat flour and Wheat products (g/d)																				
Mean	76.2	67.6	71.6	60.3	71.3	73.8	74.2	72.0	74.5	73.2	69.6	67.7	69.4	68.8	71.6	66.4	66.8	68.2	72.1	0.209
SE	3.4	3.7	3.6	3.5	3.7	4.2	4.8	5.0	4.7	4.6	4.7	2.1	4.5	4.4	5.0	2.6	4.6	4.8	5.5	
Potatoes and Starches (g/d)																				
Mean	43.6	44.7	38.4	42.0	46.6	42.8	40.7	44.8	43.1	40.8	38.5	36.6	34.9	44.2	37.4	36.2	37.4	41.7	39.7	0.024
SE	2.4	2.6	2.1	2.6	3.2	2.8	3.2	3.3	2.9	2.9	2.9	1.4	2.6	3.4	3.4	1.6	3.5	3.7	3.6	
Sugars and Sweeteners (g/d)																				
Mean	4.3	4.4	4.6	4.4	4.6	4.2	4.0	4.0	3.8	4.1	3.8	3.6	3.9	3.4	3.5	3.5	3.9	4.2	4.0	0.005
SE	0.2	0.2	0.3	0.3	0.3	0.4	0.4	0.5	0.3	0.4	0.3	0.2	0.4	0.3	0.3	0.2	0.4	0.4	0.5	
Legumes (g/d)																				
Mean	30.1	34.0	29.1	34.6	44.0	29.2	29.6	28.1	28.8	29.3	29.7	29.3	34.0	29.6	30.9	31.4	31.5	37.4	31.4	0.467
SE	2.0	2.1	1.8	2.3	3.1	2.1	2.4	2.4	2.3	2.7	2.7	1.3	2.9	3.1	2.7	1.6	2.8	3.7	3.6	
Nuts and Seeds (g/d)																				
Mean	1.2	1.5	1.1	1.2	1.2	1.5	0.5	1.1	0.9	1.0	0.6	1.0	0.6	0.5	0.9	1.2	1.3	0.6	1.9	0.376
SE	0.2	0.2	0.1	0.3	0.2	0.4	0.1	0.4	0.2	0.2	0.1	0.1	0.2	0.1	0.2	0.2	0.4	0.1	0.6	
Vegetables (g/d)																				
Mean	153.4	144.4	141.6	150.2	148.4	148.1	140.7	165.2	152.7	149.8	141.4	146.1	146.4	151.0	154.2	148.4	143.6	147.5	135.3	0.508
SE	5.5	5.1	5.2	6.6	5.8	6.6	5.7	7.0	6.1	6.4	5.8	3.1	6.1	6.9	7.2	3.5	6.1	8.1	7.6	
Fruits (g/d)																				
Mean	139.6	120.3	125.7	117.2	121.5	92.7	110.7	94.7	103.2	104.8	92.7	109.3	94.8	99.3	107.7	101.2	92.9	93.3	106.4	0.014
SE	6.8	5.9	6.1	6.7	7.9	6.4	7.9	7.2	7.1	7.6	7.9	4.1	7.2	7.3	7.8	4.1	6.3	6.8	9.4	
Mushrooms (g/d)																				
Mean	7.0	6.6	8.0	7.9	7.7	6.8	7.6	6.0	9.0	9.3	9.0	8.5	8.2	7.0	6.2	7.5	6.3	7.3	9.9	0.629
SE	0.5	0.5	0.7	0.8	0.8	0.7	0.8	0.7	0.9	1.0	1.2	0.4	1.0	1.0	0.7	0.4	0.6	0.8	1.3	
Algae (g/d)																				
Mean	8.2	9.5	7.3	7.7	9.3	9.3	7.0	5.6	5.7	6.5	6.9	5.6	5.2	5.8	4.6	5.5	6.4	5.3	4.4	<0.001
SE	0.8	1.0	0.8	1.0	0.8	1.7	0.9	0.8	0.7	0.8	1.1	0.4	0.8	1.0	0.6	0.5	0.8	0.7	0.7	
Fishes and Shellfishes (g/d)																				
Mean	38.1	40.1	40.5	36.2	41.6	35.2	38.4	30.7	32.4	31.3	33.9	31.5	26.4	28.9	31.8	31.7	30.6	33.0	33.5	0.005
SE	2.0	2.5	2.3	2.0	3.1	2.6	2.8	2.6	2.5	2.3	2.5	1.3	2.1	2.5	2.5	1.4	2.4	3.0	4.3	
Meats (g/d)																				
Mean	55.3	51.1	50.7	54.2	49.1	60.0	62.6	58.3	58.0	62.9	60.4	56.0	62.5	65.5	59.2	63.2	58.8	61.9	65.5	0.003
SE	2.2	2.1	2.5	2.4	2.7	2.9	3.0	3.0	2.9	3.0	3.4	1.5	3.2	3.8	3.2	1.7	3.1	3.6	4.6	
Eggs (g/d)																				
Mean	28.3	29.5	28.2	26.6	22.2	21.6	27.6	24.8	25.3	22.5	25.1	22.4	23.7	24.1	20.6	22.2	20.4	22.3	22.7	0.005
SE	1.4	1.4	2.0	1.7	1.6	1.4	1.7	1.7	1.6	1.5	2.0	0.9	1.7	1.9	1.6	0.9	1.5	2.0	2.6	
Milks (g/d)																				
Mean	243.1	240.9	228.4	241.5	234.5	226.8	207.7	202.6	195.6	207.4	225.8	214.0	212.8	194.5	236.5	199.0	215.0	202.4	233.6	0.038
SE	9.6	9.3	9.3	11.5	10.7	10.6	10.4	11.6	11.1	10.8	10.8	5.5	13.3	11.4	13.1	6.5	11.5	12.6	18.3	
Fats and Oils (g/d)																				
Mean	8.6	7.9	8.4	7.6	7.4	7.2	8.4	7.7	6.6	7.6	8.2	6.9	7.0	7.8	7.7	6.9	7.5	6.5	6.6	0.01
SE	0.4	0.3	0.3	0.3	0.4	0.4	0.4	0.4	0.4	0.4	0.5	0.2	0.4	0.5	0.4	0.2	0.5	0.5	0.6	
Confectioneries (g/d)																				
Mean	39.0	36.5	34.4	36.7	28.6	36.5	37.4	33.9	32.4	31.3	35.6	36.3	30.2	34.7	31.4	33.1	30.2	25.1	17.4	0.004
SE	2.5	2.5	2.5	3.1	2.8	2.6	2.9	3.1	2.7	2.6	2.9	1.6	2.8	3.1	3.1	1.7	2.8	2.6	2.3	
Beverages (g/d)																				
Mean	162.8	152.7	174.6	198.0	172.3	217.1	241.7	191.4	185.0	220.4	233.7	203.6	189.6	220.0	298.9	225.7	216.9	260.2	237.8	0.003
SE	9.3	11.0	9.6	13.3	12.8	13.5	14.6	14.0	12.1	14.5	17.3	7.1	13.4	16.6	17.9	8.3	15.0	17.7	23.4	
Seasonings and Spices (g/d)																				
Mean	40.7	40.7	38.4	43.3	44.4	46.8	48.2	53.9	53.3	46.3	46.7	47.1	51.2	36.5	48.0	52.9	41.9	32.1	37.1	0.914
SE	2.1	2.3	2.0	3.1	3.3	2.8	3.1	4.0	3.6	3.1	3.3	1.7	4.1	2.5	3.6	2.4	3.2	1.8	3.0	

SE, standard error

Animal-based foods refer to fish and shellfish, meats, eggs, milk, butter, and animal fats and oils, while plant-based foods include cereals, potatoes and starches, sugars and sweeteners, legumes, nuts and seeds, vegetables, fruits, mushrooms, algae, margarine, vegetable fats and oils, other fats and oils, confectionery, beverages, seasonings and spices.

Table S2. Trends in food consumption in young girls aged 1–6 years from 2001 to 2019.

	2001	2002	2003	2004	2005	2006	2007	2008	2009	2010	2011	2012	2013	2014	2015	2016	2017	2018	2019	$P_{for\ trend}$
n	401	341	292	257	234	283	245	217	229	225	185	799	177	164	171	633	176	208	130	
Total food weight (g/d)																				
Mean	1,198.8	1,233.1	1,192.6	1,268.4	1,252.4	1,244.3	1,200.9	1,118.0	1,159.9	1,165.9	1180.4	1,161.1	1,178.6	1106.0	1233.0	1180.5	1176.1	1134.2	1135.2	0.011
SE	20.3	21.3	21.5	23.6	23.8	22.7	23.2	22.5	23.5	23.5	28.3	12.2	28.1	25.6	26.7	13.8	26.4	22.2	29.1	
Animal-based foods (g/d)																				
Mean	347.1	346.6	326.4	348.5	325.1	309.6	313.5	276.5	295.8	300.4	309.1	298.6	318.9	273.0	295.4	301.5	284.9	287.0	299.7	0.002
SE	9.3	9.3	9.8	10.9	10.9	10.3	12.7	10.6	11.1	12.3	13.4	5.9	13.7	11.2	11.4	6.4	12.6	11.1	14.9	
Plant-based foods (g/d)																				
Mean	851.7	886.5	866.2	919.9	927.3	934.7	887.4	841.5	864.1	865.6	871.3	862.5	859.7	833.0	937.7	879.0	891.2	847.2	835.4	0.237
SE	16.9	18.3	17.7	21.4	20.9	20.1	19.3	19.4	20.6	20.1	24.5	10.7	23.1	22.2	25.5	11.5	22.7	20.3	25.0	
Cereals (g/d)																				
Mean	259.1	257.1	256.8	257.4	271.9	244.7	241.1	243.6	243.2	242.6	258.2	252.9	249.3	246.3	257.8	260.3	256.6	248.3	249.5	0.588
SE	4.7	5.1	5.1	5.9	6.8	4.9	6.2	5.9	5.7	6.1	7.0	3.1	6.4	6.4	6.3	3.1	6.1	5.9	7.3	
Rice and Rice products (g/d)																				
Mean	190.0	187.4	188.7	190.7	204.6	176.4	167.6	176.4	176.9	167.6	183.2	189.2	182.0	181.2	191.4	192.7	194.8	191.7	188.3	0.362
SE	4.7	5.0	5.0	5.6	6.4	5.4	5.6	6.4	6.0	5.8	6.6	3.1	7.2	6.1	6.7	3.3	6.6	6.1	7.2	
Wheat flour and Wheat products (g/d)																				
Mean	65.2	66.7	65.5	65.3	59.3	66.2	68.3	62.6	62.5	71.2	71.8	62.1	64.7	62.6	63.5	64.3	61.0	52.5	58.3	0.023
SE	2.9	3.5	3.4	3.8	3.8	3.4	4.5	3.8	3.8	4.4	4.8	2.0	4.4	4.5	5.2	2.4	4.1	3.7	4.1	
Potatoes and Starches (g/d)																				
Mean	41.0	49.3	41.0	45.0	43.5	44.3	34.5	34.7	41.6	38.9	40.6	40.1	36.0	27.5	37.1	35.5	37.4	38.1	33.2	0.004
SE	2.1	2.9	2.6	2.7	3.0	2.9	2.6	2.9	3.1	3.4	3.5	1.8	3.3	2.5	3.8	1.4	2.9	2.9	3.7	
Sugars and Sweeteners (g/d)																				
Mean	4.7	4.4	3.9	4.0	3.6	3.6	3.5	4.3	4.6	3.9	3.8	3.6	4.0	3.1	3.8	3.6	3.3	3.4	4.0	0.047
SE	0.3	0.3	0.2	0.2	0.2	0.3	0.3	0.4	0.4	0.3	0.3	0.2	0.4	0.4	0.4	0.2	0.3	0.3	0.4	
Legumes (g/d)																				
Mean	32.3	31.4	27.4	32.1	38.0	33.4	27.3	28.8	24.5	29.6	25.2	28.5	28.3	37.9	31.4	32.5	28.5	34.0	30.7	0.953
SE	1.8	1.7	1.7	2.5	2.9	2.3	2.3	2.4	2.3	2.4	2.9	1.5	2.8	4.6	2.8	1.4	2.5	2.8	3.5	
Nuts and Seeds (g/d)																				
Mean	1.0	1.4	1.1	1.3	1.8	0.9	0.8	1.0	1.6	1.4	1.3	0.9	0.7	1.2	0.5	1.1	0.8	0.5	1.2	0.072
SE	0.1	0.3	0.3	0.2	0.4	0.2	0.2	0.3	0.3	0.4	0.3	0.1	0.2	0.3	0.1	0.1	0.2	0.1	0.3	
Vegetables (g/d)																				
Mean	143.4	141.3	141.9	138.1	155.1	154.9	139.4	141.9	147.8	140.9	141.0	150.7	142.5	146.6	158.5	149.5	145.8	141.4	123.8	0.786
SE	4.5	4.7	4.9	5.3	5.9	5.2	6.4	6.3	6.9	5.6	6.3	3.1	6.4	7.5	8.0	3.4	7.0	7.1	6.5	
Fruits (g/d)																				
Mean	130.2	119.8	122.1	126.1	117.5	96.7	107.3	103.0	102.4	102.5	89.5	100.7	89.8	99.1	80.5	96.4	79.0	88.0	82.5	<0.001
SE	8.0	6.1	6.8	9.2	7.9	6.4	7.1	7.9	7.3	7.5	7.5	3.9	7.8	7.5	6.4	3.8	6.3	6.8	7.7	
Mushrooms (g/d)																				
Mean	7.5	8.2	8.3	7.3	9.5	8.1	8.0	7.3	8.2	8.9	6.1	7.3	8.6	7.3	7.9	6.9	7.3	7.2	7.0	0.017
SE	0.5	0.7	0.8	0.9	0.9	0.7	1.0	0.7	1.0	1.0	0.7	0.4	0.9	0.8	0.8	0.4	0.9	0.8	0.9	
Algae (g/d)																				
Mean	7.6	9.7	8.3	6.8	8.5	7.7	4.5	4.7	5.9	6.9	6.4	5.2	5.2	4.7	4.5	7.3	5.8	4.6	6.9	0.023
SE	0.6	0.9	0.9	0.6	0.9	1.0	0.6	0.6	0.8	1.0	0.9	0.4	1.0	0.8	0.7	0.6	0.7	0.6	1.3	
Fishes and Shellfishes (g/d)																				
Mean	38.9	41.4	35.2	32.7	37.3	36.4	33.4	31.0	31.9	26.2	31.4	30.1	26.3	23.3	29.1	27.4	30.1	27.2	26.6	<0.001
SE	1.9	2.3	2.1	2.1	2.6	2.6	2.4	2.5	2.2	2.2	2.7	1.2	2.3	2.2	2.5	1.2	2.6	2.3	2.7	
Meats (g/d)																				
Mean	47.9	51.6	50.7	49.4	58.7	51.8	60.9	52.9	53.6	52.2	52.7	53.6	57.3	57.6	55.4	60.1	58.0	59.1	61.3	0.002
SE	2.0	2.2	2.2	2.4	3.0	2.3	3.2	2.9	2.6	2.7	3.2	1.3	2.8	3.4	3.1	1.7	4.0	3.0	3.6	
Eggs (g/d)																				
Mean	26.8	26.3	25.6	31.1	24.9	25.6	26.9	22.4	22.3	24.0	25.1	22.2	22.8	20.1	23.6	22.8	21.6	22.2	17.2	0.001
SE	1.3	1.4	1.5	1.7	1.6	1.5	1.8	1.8	1.5	1.6	1.9	0.8	1.8	1.6	1.9	1.0	1.8	2.0	2.0	
Milks (g/d)																				
Mean	232.3	226.0	214.0	234.1	203.2	194.7	191.0	169.3	187.1	196.9	198.5	191.7	211.6	171.3	186.3	190.2	174.3	177.7	194.0	0.005
SE	8.4	8.4	9.0	10.3	10.2	9.6	11.5	9.5	10.6	11.6	12.1	5.5	12.9	10.1	10.2	5.8	11.9	10.8	14.8	
Fats and Oils (g/d)																				
Mean	7.7	7.8	7.7	8.1	7.7	6.6	7.4	6.0	6.4	6.9	8.0	6.2	6.5	6.5	6.9	6.4	6.6	5.8	6.3	0.005
SE	0.3	0.3	0.3	0.4	0.4	0.3	0.5	0.4	0.4	0.4	0.5	0.2	0.4	0.4	0.4	0.2	0.5	0.4	0.5	
Confectioneries (g/d)																				
Mean	34.3	33.6	30.4	34.2	34.1	37.0	34.3	28.0	27.2	30.4	33.0	30.5	28.8	32.8	27.0	28.1	24.4	24.9	28.4	0.003
SE	2.3	2.5	2.5	2.9	3.4	2.9	2.9	2.3	2.5	2.5	3.1	1.4	2.7	3.2	2.5	1.3	2.6	2.4	3.4	
Beverages (g/d)																				
Mean	147.4	183.9	164.8	209.5	183.5	236.5	225.9	178.9	197.5	205.1	207.3	189.6	216.2	177.4	274.8	197.6	251.6	222.7	233.9	0.021
SE	8.9	11.2	10.8	13.7	12.2	13.5	12.4	13.5	13.0	13.2	16.4	6.9	16.6	12.3	17.3	7.7	16.2	14.7	18.3	
Seasonings and Spices (g/d)																				
Mean	36.8	39.7	38.9	40.6	41.4	48.9	44.1	52.7	46.4	43.5	43.6	47.3	44.6	43.1	47.8	54.9	45.2	29.2	28.6	0.453
SE	1.8	2.4	2.3	2.5	2.4	2.4	2.8	3.6	3.5	2.9	3.2	2.1	3.4	3.5	4.5	2.0	3.5	1.1	1.9	

Table S3. Trends in food consumption in schoolboys aged 7–14 years from 2001 to 2019.

	2001	2002	2003	2004	2005	2006	2007	2008	2009	2010	2011	2012	2013	2014	2015	2016	2017	2018	2019	$P_{for\ trend}$
n	591	452	472	419	365	422	392	367	377	390	352	1271	314	320	315	1045	267	273	250	
Total food weight (g/d)																				
Mean	1,936.1	1,926.6	1,917.4	1,930.4	1,950.2	1,916.2	1,928.8	1,906.2	1,885.7	1,853.5	1854.7	1,924.9	1,888.4	1,899.0	2,010.5	1,944.5	1,980.1	1,903.4	1,873.3	0.449
SE	26.4	25.3	26.4	26.0	27.7	23.4	27.0	30.2	26.2	26.4	28.5	13.8	29.8	29.7	34.0	17.2	34.2	34.9	37.2	
Animal-based foods (g/d)																				
Mean	569.5	566.2	540.0	553.9	544.3	561.3	532.9	511.0	502.1	508.5	514.0	541.6	524.9	494.5	522.2	526.3	534.8	524.4	521.4	0.015
SE	10.0	11.6	10.1	12.1	12.5	10.0	10.9	10.3	10.0	9.8	10.3	5.7	12.0	11.2	11.4	5.8	13.7	11.9	12.3	
Plant-based foods (g/d)																				
Mean	1,366.7	1,360.4	1,377.3	1,376.4	1,405.9	1,354.8	1,395.9	1,395.2	1,383.6	1,345.0	1340.7	1,383.3	1,363.6	1,404.6	1,488.4	1,418.1	1,445.2	1,379.0	1,351.9	0.295
SE	21.3	21.2	22.9	21.1	23.3	21.6	23.6	27.5	23.8	23.5	26.2	12.8	26.7	26.6	30.4	15.7	29.5	30.8	33.5	
Cereals (g/d)																				
Mean	487.0	478.9	479.9	481.4	486.7	457.0	460.9	465.9	446.6	466.8	458.2	476.2	474.4	499.2	474.0	484.2	476.5	459.7	463.3	0.316
SE	7.6	8.8	9.0	8.4	8.3	9.3	9.0	8.6	8.5	8.7	8.9	4.7	10.8	10.6	10.6	5.7	11.5	11.4	11.0	
Rice and Rice products (g/d)																				
Mean	358.7	356.8	358.0	361.7	364.5	333.1	347.2	351.2	337.3	355.2	343.7	374.0	360.1	373.7	363.5	383.1	377.4	365.6	367.8	0.015
SE	7.3	8.8	7.9	8.6	7.9	9.0	9.7	8.8	8.6	9.2	9.1	5.2	11.2	10.5	11.4	5.9	11.7	11.6	12.0	
Wheat flour and Wheat products (g/d)																				
Mean	124.2	116.9	118.9	115.3	115.3	107.8	107.8	106.4	99.5	102.3	108.7	96.3	103.9	121.5	102.4	95.3	90.4	88.5	85.7	0.001
SE	3.8	4.4	4.5	3.6	4.2	4.9	5.1	5.4	4.8	4.7	5.9	2.6	5.2	7.3	5.3	2.8	5.7	5.5	5.8	
Potatoes and Starches (g/d)																				
Mean	88.0	80.8	81.1	80.6	79.5	69.6	64.2	64.3	65.6	57.1	60.3	65.2	60.4	57.9	56.8	65.4	56.4	62.5	54.0	<0.001
SE	2.9	2.9	2.8	2.7	3.3	3.3	3.0	3.4	3.2	2.9	2.9	1.6	2.9	3.0	3.0	2.0	3.5	3.8	3.3	
Sugars and Sweeteners (g/d)																				
Mean	7.1	6.5	6.2	6.8	6.9	5.9	6.0	6.0	5.3	5.2	5.5	5.5	5.9	5.1	5.6	5.6	6.4	6.5	6.0	0.087
SE	0.3	0.3	0.3	0.4	0.4	0.4	0.4	0.4	0.4	0.4	0.3	0.2	0.4	0.4	0.5	0.2	0.5	0.5	0.7	
Legumes (g/d)																				
Mean	56.1	49.4	51.8	56.3	54.5	43.3	41.9	46.2	37.0	39.2	37.9	48.2	47.6	49.2	50.0	45.3	64.1	53.7	45.3	0.582
SE	2.0	2.1	2.2	2.5	2.5	2.4	2.4	2.7	2.3	2.3	2.5	1.5	2.9	3.5	3.2	1.4	4.4	3.6	3.0	
Nuts and Seeds (g/d)																				
Mean	2.0	2.8	2.1	2.2	2.3	1.4	2.0	1.8	1.4	1.8	1.8	1.4	1.5	1.2	2.0	1.9	1.8	1.6	1.7	0.017
SE	0.1	0.3	0.1	0.2	0.2	0.2	0.2	0.3	0.2	0.2	0.3	0.1	0.2	0.2	0.2	0.1	0.3	0.3	0.2	
Vegetables (g/d)																				
Mean	254.3	240.9	242.1	227.5	242.5	251.0	248.7	251.5	242.4	235.0	240.7	249.2	239.9	247.7	259.3	248.6	260.6	238.4	247.9	0.64
SE	5.5	5.6	5.8	5.7	6.1	6.5	6.1	7.4	6.3	5.7	7.1	3.4	6.4	6.9	7.2	3.6	7.7	7.6	8.2	
Fruits (g/d)																				
Mean	131.5	130.5	113.7	120.4	112.3	90.5	93.9	96.4	110.5	86.1	88.3	93.3	87.0	85.6	80.7	73.9	95.4	74.3	75.0	<0.001
SE	5.0	6.7	5.3	6.6	6.2	5.3	6.1	6.1	7.3	5.8	6.5	3.5	6.5	6.7	6.8	3.3	8.7	6.2	6.2	
Mushrooms (g/d)																				
Mean	11.3	11.1	9.9	8.9	11.5	13.1	13.5	13.9	12.5	14.4	12.4	13.1	15.0	14.2	12.8	12.9	14.5	12.0	12.6	0.034
SE	0.7	0.8	0.7	0.7	1.1	1.0	1.1	1.1	0.9	0.9	1.0	0.5	1.2	1.1	0.9	0.5	1.3	1.2	1.1	
Algae (g/d)																				
Mean	11.8	13.5	11.5	11.4	10.6	12.3	10.6	8.2	8.2	7.8	8.1	8.4	8.4	7.6	8.6	8.3	8.3	6.3	4.9	0.001
SE	0.7	1.0	0.7	1.0	0.6	1.6	1.1	0.7	0.7	0.8	0.8	0.5	0.8	1.0	0.9	0.4	0.9	0.6	0.6	
Fishes and Shellfishes (g/d)																				
Mean	70.1	62.3	60.1	59.4	60.5	58.0	56.0	56.3	56.8	58.4	52.2	53.6	50.7	46.0	46.6	48.5	43.9	46.1	46.1	<0.001
SE	2.7	2.6	2.6	2.9	2.8	2.8	2.6	2.8	2.9	3.3	3.0	1.5	3.3	2.8	2.6	1.5	2.7	3.2	3.3	
Meats (g/d)																				
Mean	91.5	95.7	93.3	88.8	98.4	106.3	108.8	100.0	107.8	103.8	105.5	110.9	115.3	124.2	112.0	123.1	123.7	115.4	112.2	<0.001
SE	2.8	3.2	3.4	3.0	3.7	3.3	3.8	3.8	3.9	3.5	3.5	2.1	4.3	6.0	4.2	2.1	5.1	5.3	4.8	
Eggs (g/d)																				
Mean	39.3	41.1	42.2	35.6	38.2	36.4	36.5	38.4	33.3	35.1	35.4	34.7	32.7	33.1	35.8	30.6	31.1	36.3	34.2	0.002
SE	1.6	1.5	1.8	1.5	1.7	1.5	1.7	1.8	1.7	1.6	1.7	1.0	1.9	1.8	1.8	0.9	2.2	2.3	2.3	
Milks (g/d)																				
Mean	367.0	365.6	343.0	368.8	345.5	359.1	330.1	315.0	302.7	310.1	319.8	341.1	325.0	290.3	326.4	323.0	334.9	325.2	328.3	0.019
SE	8.8	10.3	9.3	11.1	11.4	9.6	9.8	9.4	9.6	8.8	9.1	5.4	10.1	9.0	10.1	5.2	12.1	11.4	11.1	
Fats and Oils (g/d)																				
Mean	12.1	12.6	12.4	12.7	11.9	11.1	11.8	10.2	11.0	10.8	11.3	11.2	10.9	12.3	11.5	11.2	11.5	10.1	9.1	0.011
SE	0.3	0.4	0.4	0.5	0.4	0.4	0.4	0.4	0.5	0.4	0.5	0.3	0.5	0.5	0.5	0.3	0.6	0.5	0.5	
Confectioneries (g/d)																				
Mean	41.0	42.1	38.9	35.8	39.4	38.0	41.9	38.6	40.6	35.8	36.0	38.2	37.5	34.7	37.4	37.4	40.3	38.3	35.9	0.056
SE	2.5	2.7	2.7	2.8	3.2	2.4	3.2	2.7	3.0	2.6	2.7	1.6	3.4	2.7	3.2	1.5	3.5	3.6	2.8	
Beverages (g/d)																				
Mean	209.3	235.2	259.3	266.6	283.0	283.0	315.9	297.6	307.3	307.4	299.1	299.3	288.4	318.2	422.4	342.0	335.1	360.5	342.7	<0.001
SE	11.9	12.1	12.8	12.6	16.2	14.3	15.7	20.4	16.2	15.4	17.8	8.9	18.0	18.5	19.4	10.5	20.3	20.4	25.1	
Seasonings and Spices (g/d)																				
Mean	56.7	57.6	58.5	57.0	56.4	71.0	77.7	88.2	83.0	68.7	73.7	75.3	87.7	72.5	68.7	82.8	75.6	56.5	54.5	0.48
SE	1.9	2.1	2.5	2.9	3.1	2.7	3.4	4.1	3.6	2.9	3.2	2.0	4.5	3.4	3.4	2.1	3.9	2.7	2.2	

Table S4. Trends in food consumption in schoolgirls aged 7–14 years from 2001 to 2019.

	2001	2002	2003	2004	2005	2006	2007	2008	2009	2010	2011	2012	2013	2014	2015	2016	2017	2018	2019	$P_{for\ trend}$
n	580	464	467	351	376	393	403	337	382	349	368	1285	295	300	282	943	245	244	204	
Total food weight (g/d)																				
Mean	1,699.0	1,742.5	1,696.4	1,754.9	1,721.0	1,726.3	1,722.0	1,660.4	1,726.8	1,691.4	1732.5	1,717.6	1,686.2	1703.2	1827.8	1705.1	1796.2	1648.2	1651.5	0.381
SE	19.9	22.6	21.6	23.9	22.6	21.8	24.2	25.1	20.8	22.9	24.7	12.0	24.6	23.0	28.1	13.5	28.7	27.8	30.9	
Animal-based foods (g/d)																				
Mean	481.3	481.2	454.1	477.6	465.6	494.7	466.1	450.5	452.8	457.4	463.1	459.8	454.4	457.5	449.9	461.0	485.7	454.2	456.7	0.131
SE	8.5	9.3	8.9	10.1	8.7	9.2	8.8	9.9	9.5	9.4	9.1	4.6	9.2	8.9	10.1	5.1	10.4	11.4	10.8	
Plant-based foods (g/d)																				
Mean	1,217.7	1,261.3	1,242.3	1,277.3	1,255.5	1,231.6	1,255.9	1,210.0	1,274.1	1,234.0	1269.3	1,257.8	1,231.8	1245.6	1378.0	1244.1	1310.5	1194.0	1194.9	0.772
SE	15.7	19.1	18.1	21.6	20.3	18.9	21.7	21.9	19.1	20.4	21.9	11.2	21.8	21.4	25.2	12.1	25.9	23.1	28.2	
Cereals (g/d)																				
Mean	404.2	412.0	398.2	406.9	400.6	395.3	388.3	384.0	383.8	398.5	396.3	396.2	395.1	395.4	406.5	396.5	410.9	390.5	387.2	0.204
SE	5.3	5.7	6.2	6.5	5.7	6.6	6.6	6.6	6.6	6.7	6.1	3.6	7.6	7.2	8.4	3.8	7.9	7.8	8.7	
Rice and Rice products (g/d)																				
Mean	285.7	304.7	290.0	286.8	292.3	287.8	278.5	277.9	287.2	295.7	292.2	298.1	291.2	291.6	309.9	299.9	312.0	288.9	289.4	0.117
SE	5.1	5.4	5.9	6.7	5.9	7.1	7.3	7.7	7.3	7.1	7.0	3.8	8.3	8.1	9.2	4.5	8.4	8.8	9.2	
Wheat flour and Wheat products (g/d)																				
Mean	114.7	105.5	105.5	116.5	106.8	101.7	105.6	99.4	87.8	96.0	99.4	92.2	97.8	99.6	90.9	89.7	90.1	95.6	89.8	0.001
SE	3.5	3.5	3.5	4.3	3.6	4.7	4.6	4.9	4.3	4.5	4.8	2.5	5.1	6.1	5.1	2.6	5.7	5.6	6.2	
Potatoes and Starches (g/d)																				
Mean	74.9	73.3	72.0	75.9	75.2	67.4	56.9	54.1	59.4	57.7	64.3	60.8	54.4	59.1	55.7	59.2	63.6	53.9	51.7	0.002
SE	2.2	2.4	2.9	3.0	3.3	2.9	2.6	2.9	2.9	2.9	3.1	1.6	3.1	3.0	3.4	2.0	4.6	3.2	3.5	
Sugars and Sweeteners (g/d)																				
Mean	6.0	6.1	6.1	6.2	6.4	5.7	5.9	4.5	5.8	5.5	5.4	5.7	5.6	5.5	5.0	5.4	7.2	4.9	5.3	0.015
SE	0.2	0.3	0.3	0.3	0.3	0.3	0.4	0.3	0.4	0.5	0.3	0.2	0.4	0.4	0.3	0.2	0.8	0.4	0.5	
Legumes (g/d)																				
Mean	54.1	49.5	49.7	51.9	52.6	38.7	40.8	45.3	41.7	38.5	39.2	45.4	46.1	43.7	43.9	43.4	47.8	49.7	42.2	0.182
SE	2.1	2.1	2.2	2.5	2.3	2.2	2.5	3.0	2.7	2.5	2.6	1.4	2.9	2.9	2.7	1.5	3.5	3.6	3.4	
Nuts and Seeds (g/d)																				
Mean	2.0	1.9	2.1	2.7	2.4	1.6	1.7	1.5	2.0	1.9	1.9	1.9	1.5	1.7	2.0	2.2	2.0	1.2	1.8	0.183
SE	0.1	0.2	0.2	0.4	0.2	0.2	0.2	0.2	0.2	0.2	0.3	0.1	0.3	0.2	0.5	0.2	0.3	0.3	0.4	
Vegetables (g/d)																				
Mean	236.5	227.0	235.5	225.2	246.1	249.2	238.9	225.8	237.7	224.3	236.8	240.9	234.5	243.5	231.7	234.7	233.5	229.4	232.8	0.598
SE	4.8	5.4	5.7	6.0	6.5	6.9	6.1	6.5	6.2	5.8	5.7	3.1	6.3	6.7	6.8	3.3	7.7	7.7	8.1	
Fruits (g/d)																				
Mean	133.7	132.9	122.9	120.7	127.1	94.5	96.0	99.4	108.7	99.1	77.1	87.8	91.9	91.2	81.2	82.1	87.3	71.1	72.5	<0.001
SE	5.8	5.9	5.7	6.4	6.9	5.2	6.4	7.1	6.7	6.2	5.2	3.0	6.2	7.0	6.6	3.1	7.5	5.9	6.0	
Mushrooms (g/d)																				
Mean	11.5	10.5	11.0	10.8	12.6	11.7	12.7	15.3	13.9	13.8	12.0	12.2	14.8	13.6	12.4	11.9	14.1	12.6	17.0	0.012
SE	0.8	0.7	0.9	0.8	1.0	0.8	0.9	1.2	1.0	1.0	0.9	0.5	1.3	1.1	1.0	0.5	1.3	1.4	1.7	
Algae (g/d)																				
Mean	11.6	12.0	11.6	10.8	10.8	9.3	8.0	7.1	9.2	8.7	9.3	7.4	7.5	8.5	6.4	8.1	8.1	7.1	7.0	0.001
SE	0.7	0.8	0.8	0.7	0.7	0.8	0.8	0.7	0.8	0.9	0.9	0.4	0.8	1.1	0.7	0.5	1.0	0.8	0.9	
Fishes and Shellfishes (g/d)																				
Mean	59.9	56.3	61.2	55.7	55.1	60.4	50.4	51.0	51.9	48.3	49.3	46.1	45.8	48.3	45.0	43.4	48.7	41.1	44.2	<0.001
SE	2.3	2.3	2.6	2.8	2.7	2.9	2.6	2.9	2.6	2.5	2.7	1.3	2.8	2.7	2.8	1.4	3.1	2.7	3.0	
Meats (g/d)																				
Mean	81.1	81.3	81.9	77.8	83.0	96.6	95.8	84.8	99.5	92.0	95.8	96.7	98.0	97.2	89.2	99.1	98.9	101.8	107.5	<0.001
SE	2.5	2.7	3.1	2.7	2.9	3.3	3.0	3.2	3.5	3.4	3.1	1.7	3.4	3.5	3.1	1.8	3.8	4.4	4.7	
Eggs (g/d)																				
Mean	37.2	38.9	37.3	32.2	34.6	34.9	33.7	34.1	33.9	30.1	31.9	30.5	30.0	31.8	33.7	29.5	31.5	32.0	32.6	0.003
SE	1.3	1.4	1.4	1.5	1.5	1.7	1.5	1.8	1.6	1.8	1.7	0.8	1.7	1.9	2.0	0.9	2.1	2.0	2.4	
Milks (g/d)																				
Mean	301.7	303.3	272.3	310.4	291.3	301.2	284.8	279.2	266.2	285.7	284.7	285.1	279.6	279.3	280.3	287.4	305.2	278.4	271.3	0.109
SE	7.6	8.4	7.8	9.7	8.0	8.7	8.3	9.1	8.7	8.6	7.9	4.2	8.7	8.4	9.1	5.0	9.7	9.9	10.1	
Fats and Oils (g/d)																				
Mean	11.2	11.2	11.2	10.9	11.1	11.4	10.3	9.0	10.0	9.8	10.2	10.4	9.9	9.5	10.7	10.6	11.5	9.3	9.0	0.035
SE	0.3	0.4	0.4	0.4	0.4	0.5	0.4	0.4	0.4	0.4	0.4	0.2	0.5	0.4	0.4	0.3	0.6	0.5	0.5	
Confectioneries (g/d)																				
Mean	39.0	42.6	37.2	39.2	34.2	40.6	45.2	38.3	39.1	35.9	38.3	35.9	34.6	36.2	36.4	37.2	38.1	30.0	35.9	0.019
SE	2.3	2.8	2.7	3.0	3.0	2.8	3.0	3.0	2.4	2.6	2.6	1.3	2.6	2.7	3.0	1.5	3.0	2.5	3.2	
Beverages (g/d)																				
Mean	183.0	231.1	225.6	252.9	214.2	231.1	272.7	243.4	276.8	266.2	307.3	281.8	266.0	276.6	419.5	282.8	320.5	284.4	282.3	<0.001
SE	8.3	11.7	10.3	13.1	12.5	11.6	13.7	13.3	13.5	14.5	15.7	7.7	15.0	16.9	18.4	8.6	17.5	17.2	20.6	
Seasonings and Spices (g/d)																				
Mean	51.4	52.7	55.4	57.5	56.1	69.7	72.8	75.8	76.8	67.4	61.4	72.7	70.9	62.2	68.0	71.7	67.4	50.7	51.4	0.716
SE	1.6	2.4	2.5	3.0	2.4	2.9	3.2	3.4	3.3	2.8	2.1	1.8	3.5	2.5	3.8	1.9	3.6	2.0	2.5	

Table S5. Trends in food consumption in adolescent boys aged 15–19 years from 2001 to 2019.

	2001	2002	2003	2004	2005	2006	2007	2008	2009	2010	2011	2012	2013	2014	2015	2016	2017	2018	2019	$P_{for\ trend}$
n	358	301	272	239	218	239	201	190	206	193	193	702	175	173	165	559	141	143	130	
Total food weight (g/d)																				
Mean	2,184.3	2,121.7	2,271.7	2,201.0	2,113.7	2,241.7	2,204.5	2,104.1	2,291.5	2,204.8	2234.5	2,197.6	2,182.1	2,076.4	2,542.7	2,110.7	2,206.3	2,200.3	2,148.2	0.789
SE	43.1	40.5	49.1	45.2	51.2	47.7	49.2	51.3	52.8	53.8	54.5	26.0	50.9	54.7	68.8	27.3	60.1	56.7	72.3	
Animal-based foods (g/d)																				
Mean	510.4	496.7	511.4	481.3	451.5	465.5	464.7	415.6	456.1	444.4	445.1	452.3	420.9	423.5	474.6	439.0	477.3	462.1	464.9	0.086
SE	16.0	16.9	18.8	18.0	19.8	17.8	19.9	17.3	18.4	19.8	19.7	8.9	16.5	18.0	20.6	9.5	21.9	21.6	22.4	
Plant-based foods (g/d)																				
Mean	1,673.9	1,624.9	1,760.3	1,719.7	1,662.1	1,776.2	1,739.8	1,688.5	1,835.4	1,760.4	1789.4	1,745.3	1,761.3	1,652.9	2,068.2	1,671.6	1,729.0	1,738.1	1,683.3	0.582
SE	36.0	33.6	42.9	38.6	43.2	41.2	42.3	45.9	47.2	47.5	48.4	23.6	45.3	48.3	60.5	23.7	52.6	48.1	61.6	
Cereals (g/d)																				
Mean	649.9	624.0	625.0	603.0	631.1	652.2	615.4	629.8	624.6	685.3	652.1	649.7	680.7	619.8	668.7	638.4	637.9	645.4	630.5	0.246
SE	14.3	13.6	15.2	12.8	17.0	14.9	16.1	16.8	16.5	19.2	15.9	8.8	20.2	21.0	21.1	8.7	20.4	18.7	20.3	
Rice and Rice products (g/d)																				
Mean	518.0	503.5	496.0	480.1	511.2	547.3	502.0	516.4	499.8	559.4	520.5	517.5	548.5	483.0	542.7	529.8	535.1	523.3	518.2	0.052
SE	13.7	13.8	16.2	13.2	17.9	15.9	15.5	19.0	17.1	19.6	16.9	9.4	20.8	21.4	21.3	9.2	21.4	21.1	21.3	
Wheat flour and Wheat products (g/d)																				
Mean	121.6	115.6	123.4	114.4	115.3	100.4	112.2	106.8	120.9	117.0	127.2	127.9	129.1	131.5	120.0	103.8	95.4	117.0	105.8	0.716
SE	6.2	7.8	7.1	7.2	7.8	7.3	8.5	8.9	9.0	8.6	9.9	4.9	10.2	9.3	8.4	4.7	9.5	9.6	10.1	
Potatoes and Starches (g/d)																				
Mean	57.4	64.3	67.3	64.5	59.9	62.0	58.6	58.7	59.1	60.0	63.5	57.7	59.2	49.5	61.1	56.8	62.9	57.6	68.1	0.476
SE	3.2	4.1	4.9	4.5	5.1	4.6	4.8	5.7	4.4	5.3	4.9	2.6	4.9	4.3	5.2	2.5	5.6	6.7	9.5	
Sugars and Sweeteners (g/d)																				
Mean	6.1	6.7	5.9	6.2	5.9	7.1	6.4	7.2	7.2	5.9	6.9	6.8	7.1	8.0	6.1	6.9	6.7	7.5	6.2	0.134
SE	0.5	0.6	0.5	0.6	0.5	0.7	0.7	0.7	0.7	0.5	0.7	0.4	0.7	0.8	0.6	0.4	0.9	0.8	0.8	
Legumes (g/d)																				
Mean	48.2	49.6	43.2	47.6	46.1	50.2	43.6	42.1	58.4	39.9	49.9	49.9	43.6	44.2	55.6	49.7	47.7	47.7	40.8	0.994
SE	3.2	3.7	3.9	3.9	4.2	4.3	4.2	4.0	5.1	4.7	5.4	2.6	4.4	4.3	6.5	2.5	5.6	4.9	6.2	
Nuts and Seeds (g/d)																				
Mean	1.6	2.3	1.6	1.8	1.1	1.1	1.2	1.5	1.1	1.7	1.3	1.6	3.2	1.6	2.6	1.9	0.5	1.2	1.0	0.615
SE	0.3	0.4	0.8	0.4	0.2	0.3	0.2	0.3	0.3	0.4	0.3	0.4	1.8	0.3	1.0	0.3	0.1	0.3	0.3	
Vegetables (g/d)																				
Mean	267.8	254.0	257.0	238.3	254.9	264.5	284.2	263.6	260.7	237.6	266.6	271.5	264.3	260.2	296.9	250.7	277.9	279.6	240.2	0.397
SE	7.8	8.6	9.0	9.9	9.0	9.3	11.5	12.9	11.9	10.5	11.9	5.8	12.3	12.5	12.3	5.9	13.5	14.4	13.9	
Fruits (g/d)																				
Mean	113.2	122.2	104.6	108.5	110.8	110.5	80.2	74.8	96.6	83.4	88.1	92.5	83.8	54.6	88.5	74.9	84.7	68.7	59.6	0.001
SE	8.1	10.4	10.1	9.7	12.5	10.9	10.8	8.2	9.9	9.8	11.5	5.6	10.3	6.5	10.7	4.6	13.1	9.0	8.9	
Mushrooms (g/d)																				
Mean	15.4	12.9	14.0	12.5	15.9	15.1	12.8	12.5	11.8	15.3	13.4	12.9	15.0	12.3	14.7	14.7	10.0	15.6	10.2	0.353
SE	1.4	1.2	1.6	1.4	1.8	1.9	1.4	1.5	1.4	1.7	1.7	0.9	1.9	1.6	2.0	1.0	1.5	2.0	1.9	
Algae (g/d)																				
Mean	11.3	10.7	9.5	9.0	8.7	11.0	10.4	7.0	8.7	10.1	7.6	8.7	8.9	8.3	7.5	8.1	8.0	7.4	8.5	0.003
SE	1.1	1.1	1.2	1.0	1.2	1.4	1.3	1.3	1.6	1.4	1.0	0.6	1.2	2.5	1.6	0.6	1.0	1.3	1.3	
Fishes and Shellfishes (g/d)																				
Mean	83.7	79.7	82.5	63.0	72.1	73.9	64.7	66.2	62.2	59.9	56.2	54.9	61.6	54.3	56.0	51.5	63.7	57.8	42.4	<0.001
SE	4.2	4.0	5.3	4.3	4.5	5.3	4.3	5.3	5.1	4.8	3.9	2.3	4.7	4.6	4.6	2.1	5.4	5.4	4.6	
Meats (g/d)																				
Mean	140.5	142.5	149.4	139.7	148.8	156.6	166.1	149.5	149.3	159.1	148.6	166.8	163.0	157.4	184.6	180.7	186.7	194.4	190.8	<0.001
SE	4.9	5.3	6.3	6.1	6.9	6.4	8.8	7.2	7.3	7.4	6.4	4.0	7.2	7.0	8.3	4.5	9.5	10.8	10.5	
Eggs (g/d)																				
Mean	51.7	50.4	56.4	49.6	48.9	53.2	53.6	47.4	49.8	52.5	52.5	50.1	47.6	55.9	56.3	50.5	53.1	55.6	60.0	0.141
SE	2.1	2.4	3.1	2.4	3.1	2.6	3.0	2.9	2.9	3.0	3.2	1.5	3.0	3.3	3.5	1.7	3.5	4.4	4.7	
Milks (g/d)																				
Mean	232.5	222.1	221.3	227.4	180.4	180.6	178.6	151.0	193.5	171.3	186.3	179.0	147.5	153.9	176.1	154.8	172.3	153.3	169.6	0.002
SE	13.6	15.0	16.5	15.5	17.0	15.2	18.1	14.4	16.2	15.9	16.9	7.6	13.9	15.7	18.0	7.7	18.2	16.0	16.8	
Fats and Oils (g/d)																				
Mean	16.6	16.7	16.9	16.9	16.2	16.4	15.8	14.5	14.9	16.0	14.0	16.6	15.0	15.7	16.9	15.2	16.1	14.7	17.1	0.359
SE	0.6	0.8	0.7	0.7	0.9	0.8	0.9	0.8	0.8	0.9	0.6	0.4	1.0	1.0	1.1	0.5	1.0	1.0	1.2	
Confectioneries (g/d)																				
Mean	39.2	39.5	37.7	40.4	28.7	33.2	36.6	34.6	36.7	31.8	30.0	34.4	34.1	30.1	31.9	28.0	27.7	26.6	34.7	0.005
SE	3.5	3.8	3.7	4.4	3.8	3.9	4.7	4.3	4.2	3.5	3.6	2.3	4.5	3.7	4.8	1.9	4.3	3.8	4.5	
Beverages (g/d)																				
Mean	371.0	338.6	477.7	463.1	388.3	458.8	467.5	441.0	549.7	474.0	498.7	454.9	443.1	460.4	732.0	439.5	464.6	497.3	504.6	0.055
SE	21.0	19.8	28.8	25.1	26.8	27.9	29.6	30.8	31.8	30.9	30.7	16.5	28.6	31.8	47.8	15.7	36.6	32.9	45.2	
Seasonings and Spices (g/d)																				
Mean	78.3	85.5	91.5	94.6	89.3	87.2	94.3	94.7	95.0	95.7	84.8	89.7	104.4	90.3	87.2	88.3	85.8	70.1	63.9	0.353
SE	3.4	4.7	5.4	6.1	5.6	4.9	6.1	6.5	5.5	5.7	5.2	3.2	7.8	5.7	5.4	3.2	6.3	3.5	3.5	

Table S6. Trends in food consumption in adolescent girls aged 15–19 years from 2001 to 2019.

	2001	2002	2003	2004	2005	2006	2007	2008	2009	2010	2011	2012	2013	2014	2015	2016	2017	2018	2019	*P* for trend
n	330	314	291	196	211	219	192	170	197	193	187	599	162	182	169	491	142	134	119	
Total food weight (g/d)																				
Mean	1,759.8	1,678.5	1,742.3	1,738.5	1,764.1	1,767.1	1,797.0	1,715.8	1,697.1	1,685.2	1693.3	1,726.6	1,692.5	1666.2	1823.3	1644.2	1731.7	1688.6	1695.5	0.112
SE	32.5	29.5	35.4	36.3	38.2	34.9	39.4	38.2	38.8	38.3	38.0	22.1	42.3	35.5	41.3	22.9	47.8	43.0	45.4	
Animal-based foods (g/d)																				
Mean	381.1	361.1	360.5	386.5	353.8	361.5	360.4	312.2	327.1	328.5	338.7	353.7	329.3	318.5	327.7	333.4	351.8	319.6	364.4	0.035
SE	11.6	11.3	11.0	16.3	11.9	12.9	14.0	11.7	13.0	12.8	12.7	8.0	12.0	13.0	14.7	7.5	15.1	14.4	16.9	
Plant-based foods (g/d)																				
Mean	1,378.7	1,317.4	1,381.8	1,352.0	1,410.3	1,405.6	1,436.6	1,403.6	1,370.0	1,356.7	1354.5	1,372.9	1,363.2	1347.6	1495.5	1310.8	1379.9	1369.0	1331.1	0.312
SE	27.7	25.2	31.7	32.7	33.3	31.8	35.5	35.6	35.0	34.1	34.2	19.7	39.2	32.8	36.8	20.5	45.5	38.5	39.5	
Cereals (g/d)																				
Mean	428.5	419.4	417.5	419.6	416.4	420.3	406.0	418.0	399.1	412.2	409.3	410.8	396.0	406.2	418.9	404.7	424.5	417.2	408.4	0.092
SE	8.4	7.9	8.7	12.2	10.7	9.1	9.9	10.2	10.3	11.0	10.4	6.0	11.8	10.1	11.5	5.8	13.7	12.0	11.4	
Rice and Rice products (g/d)																				
Mean	322.8	318.2	308.1	317.8	307.1	317.9	311.5	319.3	305.4	306.5	311.9	316.0	298.5	322.7	328.9	314.8	325.2	332.2	328.5	0.131
SE	8.7	7.9	8.6	12.2	10.8	10.3	10.4	11.0	10.9	11.5	10.9	6.3	12.8	11.3	11.9	6.5	15.0	14.2	11.8	
Wheat flour and Wheat products (g/d)																				
Mean	102.5	94.8	102.0	99.4	103.8	100.5	90.4	88.4	91.5	104.5	94.6	91.1	94.5	80.2	87.7	84.8	94.7	82.3	70.9	0.002
SE	5.4	5.0	6.1	7.3	7.5	7.1	6.4	7.8	6.6	8.0	7.3	3.8	7.5	6.9	7.4	4.0	8.1	8.3	8.6	
Potatoes and Starches (g/d)																				
Mean	58.2	55.2	51.3	64.1	60.8	52.3	51.9	53.7	52.2	47.1	59.2	51.9	49.7	57.2	44.6	48.6	51.5	52.2	53.9	0.087
SE	3.5	3.4	3.7	4.5	5.3	3.8	4.0	5.0	4.4	3.9	4.5	2.6	5.1	4.7	3.7	2.6	5.3	5.6	5.6	
Sugars and Sweeteners (g/d)																				
Mean	6.5	6.4	5.9	6.4	6.3	7.1	6.5	5.9	6.3	6.2	6.6	6.6	5.9	6.5	6.2	5.6	5.8	5.0	6.0	0.044
SE	0.4	0.5	0.4	0.5	0.6	0.6	0.8	0.7	0.6	0.5	0.6	0.3	0.6	0.7	0.6	0.4	0.5	0.5	0.7	
Legumes (g/d)																				
Mean	43.9	38.7	37.4	47.1	49.5	39.8	42.6	37.9	46.4	43.8	45.1	38.6	48.3	46.9	47.6	37.0	49.1	50.0	40.9	0.265
SE	3.1	3.0	2.7	4.2	4.7	3.3	4.0	3.5	4.3	4.9	4.3	2.0	4.9	4.7	6.1	2.3	7.6	5.1	5.8	
Nuts and Seeds (g/d)																				
Mean	1.3	1.7	1.8	0.7	1.4	2.8	1.2	1.6	1.2	1.3	1.7	1.2	1.2	1.5	1.2	1.4	2.0	1.2	1.7	0.829
SE	0.2	0.3	0.3	0.2	0.2	0.9	0.2	0.4	0.3	0.4	0.3	0.2	0.3	0.3	0.3	0.2	0.5	0.2	0.4	
Vegetables (g/d)																				
Mean	240.0	215.3	230.2	212.2	261.9	259.4	250.4	246.7	229.5	228.0	244.5	255.3	228.4	225.6	229.7	216.6	226.4	232.3	246.9	0.733
SE	7.5	6.8	8.9	9.8	10.8	10.1	11.0	11.5	9.7	9.7	10.7	5.8	9.8	10.1	9.6	5.7	12.0	11.2	13.7	
Fruits (g/d)																				
Mean	135.1	105.9	98.2	108.1	115.9	101.7	90.1	86.0	86.9	76.8	75.7	83.0	89.1	83.7	74.6	69.9	74.4	55.0	73.6	<0.001
SE	9.1	7.7	7.5	10.5	9.7	9.1	9.7	8.8	9.2	9.6	8.2	4.9	10.3	8.6	8.8	5.0	11.3	6.9	10.5	
Mushrooms (g/d)																				
Mean	13.0	11.7	12.1	14.1	13.7	16.6	16.7	13.1	13.2	11.5	13.7	13.3	15.5	12.9	15.7	11.6	12.0	11.8	17.8	1.000
SE	1.2	1.1	1.3	1.6	1.9	2.2	1.9	1.6	1.8	1.5	1.7	1.0	2.3	1.8	1.9	0.8	1.7	1.6	3.5	
Algae (g/d)																				
Mean	11.8	8.5	8.9	9.6	8.1	7.6	10.3	10.4	8.1	6.5	8.2	8.2	8.2	7.2	9.8	8.1	8.1	7.4	6.9	0.021
SE	1.2	0.8	1.0	1.3	0.9	1.4	1.5	1.4	1.3	0.8	1.2	0.8	1.5	1.0	1.5	0.7	1.2	1.8	1.3	
Fishes and Shellfishes (g/d)																				
Mean	76.5	66.5	62.7	69.2	64.0	62.7	56.9	57.4	56.8	48.3	44.1	49.2	42.9	51.3	55.3	42.6	37.6	40.2	44.3	<0.001
SE	3.7	3.4	3.5	5.1	4.5	4.5	4.0	4.1	3.8	4.0	3.4	2.3	4.1	4.1	4.2	2.4	3.6	4.7	4.3	
Meats (g/d)																				
Mean	98.6	96.5	102.3	110.2	115.2	108.3	112.7	99.2	110.4	115.8	112.5	119.0	125.2	97.1	112.3	124.6	128.9	133.9	143.6	0.002
SE	4.1	3.5	4.2	4.8	5.3	4.8	5.2	4.8	4.8	5.4	5.0	3.1	6.3	4.8	5.6	3.3	6.6	6.9	7.8	
Eggs (g/d)																				
Mean	45.3	47.1	48.2	43.7	47.1	49.4	47.6	46.4	43.7	45.1	43.6	43.6	45.6	44.2	49.9	44.3	47.5	51.2	48.8	0.537
SE	2.1	2.1	2.6	2.6	2.7	2.7	2.5	2.6	2.4	2.7	2.4	1.5	2.9	2.6	3.3	1.6	2.7	3.3	3.8	
Milks (g/d)																				
Mean	158.7	149.2	145.4	161.9	125.7	139.2	141.5	107.9	114.4	117.7	137.3	140.8	114.0	124.4	108.4	120.7	136.4	93.1	126.6	0.008
SE	9.9	9.4	10.0	14.2	9.8	11.1	12.3	10.8	11.1	11.3	11.0	7.0	9.8	11.4	11.6	6.3	13.3	11.2	12.8	
Fats and Oils (g/d)																				
Mean	13.9	13.3	12.6	13.0	13.5	13.0	13.8	11.2	12.9	13.1	11.5	12.6	12.9	11.8	13.4	11.6	13.3	12.0	13.3	0.21
SE	0.6	0.5	0.6	0.7	0.7	0.7	0.7	0.7	0.6	0.7	0.7	0.4	0.7	0.7	0.8	0.4	0.9	0.7	1.0	
Confectioneries (g/d)																				
Mean	40.1	42.6	38.2	33.1	43.1	32.1	32.3	35.2	37.8	34.0	30.6	34.8	33.8	35.8	36.2	30.8	31.4	31.6	34.6	0.026
SE	3.3	3.4	3.6	3.6	4.6	3.6	3.7	3.9	4.4	3.7	3.6	2.4	4.5	4.6	4.0	2.3	4.8	4.3	4.8	
Beverages (g/d)																				
Mean	318.2	332.2	380.2	343.5	342.6	368.1	427.3	401.1	404.5	395.5	372.1	382.5	403.0	390.5	531.9	399.5	415.9	443.9	374.2	0.007
SE	16.8	17.4	19.6	21.6	21.2	24.9	22.9	28.4	22.7	24.1	22.6	13.9	26.7	23.7	28.2	14.4	32.6	31.2	29.6	
Seasonings and Spices (g/d)																				
Mean	70.2	68.3	78.3	72.0	73.1	75.9	76.5	78.3	69.2	76.9	69.6	75.3	72.8	63.5	67.5	66.7	67.0	50.6	53.9	0.008
SE	3.3	3.2	4.6	4.7	4.6	5.2	4.9	6.4	5.1	5.3	4.8	2.8	5.4	4.0	3.9	2.8	4.8	2.2	2.8	

Table S7. Annual percentage change (APC) in food consumption among Japanese children and adolescents by sex and age from 2001 to 2019.

Variables	Boys			Girls		
	Year	APC	Trend†	Year	APC	Trend
Animal-based foods (g/d)						
1-6 years	2001-2009	-1.56*	Decrease	2001-2008	-2.35*	Decrease
	2009-2019	0.11	Stable	2008-2019	-0.11	Stable
7-14 years	2001-2009	-1.12*	Decrease	2001-2019	-0.22	Stable
	2009-2019	0.17	Stable			
15-19 years	2001-2008	-2.23*	Decrease	2001-2019	-0.62*	Decrease
	2008-2019	0.32	Stable			
Plant-based foods (g/d)						
1-6 years	2001-2019	0.06	Stable	2001-2019	-0.14	Stable
7-14 years	2001-2019	0.18	Stable	2001-2019	0.06	Stable
15-19 years	2001-2019	0.16	Stable	2001-2019	-0.07	Stable
Cereals (g/d)						
1-6 years	2001-2019	-0.19	Stable	2001-2008	-0.84	Stable
				2008-2019	0.47	Stable
7-14 years	2001-2009	-0.93*	Stable	2001-2008	-0.64	Stable
	2009-2014	1.55*	Increase	2008-2019	0.21	Stable
	2014-2019	-1.15	Decrease			
15-19 years	2001-2019	0.17	Stable	2001-2019	-0.20*	Stable
Potatoes and Starches (g/d)						
1-6 years	2001-2019	-0.96*	Stable	2001-2019	-1.38*	Decrease
7-14 years	2001-2008	-4.39*	Decrease	2001-2019	-1.84*	Decrease
	2008-2019	-0.74	Stable			
15-19 years	2001-2019	-0.44	Stable	2001-2019	-0.84*	Stable
Sugars and Sweeteners (g/d)						
1-6 years	2001-2003	3.56	Increase	2001-2019	-0.99*	Stable
	2003-2015	-2.46*	Decrease			
	2015-2019	5.47*	Increase			
7-14 years	2001-2014	-2.06*	Decrease	2001-2019	-0.87*	Stable
	2014-2019	4.54	Increase			
15-19 years	2001-2019	0.73	Stable	2001-2012	0.24	Stable
				2012-2019	-2.68*	Decrease
Legumes (g/d)						
1-6 years	2001-2019	-0.20	Stable	2001-2019	0.01	Stable
7-14 years	2001-2009	-3.58*	Decrease	2001-2009	-3.08*	Decrease
	2009-2019	2.15	Increase	2009-2019	1.08	Increase
15-19 years	2001-2019	0.14	Stable	2001-2019	0.12	Stable
Nuts and Seeds (g/d)						
1-6 years	2001-2019	-2.52	Decrease	2001-2019	-1.59	Decrease
7-14 years	2001-2019	-1.43*	Decrease	2001-2019	-0.58	Stable
15-19 years	2001-2019	-2.03	Decrease	2001-2019	-0.21	Stable
Vegetables (g/d)						
1-6 years	2001-2019	-0.08	Stable	2001-2017	0.31	Stable
				2017-2019	-8.51*	Decrease
7-14 years	2001-2019	0.15	Stable	2001-2019	0.01	Stable
15-19 years	2001-2019	0.13	Stable	2001-2019	-0.09	Stable
Fruits (g/d)						
1-6 years	2001-2006	-5.04*	Decrease	2001-2019	-2.14*	Decrease
	2006-2019	-0.42	Stable			
7-14 years	2001-2019	-3.11*	Stable	2001-2019	-3.30*	Decrease
15-19 years	2001-2019	-2.96*	Stable	2001-2019	-3.38*	Decrease

Animal-based foods refer to fish and shellfish, meats, eggs, milk, butter, and animal fats and oils, while plant-based foods include cereals, potatoes and starches, sugars and sweeteners, legumes, nuts and seeds, vegetables, fruits, mushrooms, algae, margarine, vegetable fats and oils, other fats and oils, confectionery, beverages, seasonings and spices.

* $p < 0.05$

† Trends were defined as a decrease (below -1), stable (-1 to below 1), and an increase (above 1).

Table S7. *Cont.*

Variables	Boys				Girls		
	Year	APC	Trend†		Year	APC	Trend
Mushrooms (g/d)							
1-6 years	2001-2019	0.39	Stable		2001-2019	-0.77	Stable
7-14 years	2001-2004	-6.25	Decrease		2001-2019	0.97	Stable
	2004-2007	13.67	Increase				
	2007-2019	-0.65	Stable				
15-19 years	2001-2019	-0.25	Stable		2001-2019	-0.14	Stable
Algae (g/d)							
1-6 years	2001-2019	-3.37*	Decrease		2001-2019	-2.33*	Decrease
7-14 years	2001-2010	-4.88*	Decrease		2001-2019	-3.07*	Decrease
	2010-2017	0.29	Stable				
	2017-2019	-23.70*	Decrease				
15-19 years	2001-2019	-1.73*	Decrease		2001-2019	-1.38*	Decrease
Fishes and Shellfishes (g/d)							
1-6 years	2001-2013	-2.49*	Decrease		2001-2019	-2.30*	Decrease
	2013-2019	1.61	Increase				
7-14 years	2001-2019	-2.09*	Decrease		2001-2019	-2.02*	Decrease
15-19 years	2001-2019	-2.82*	Decrease		2001-2019	-3.22*	Decrease
Meats (g/d)							
1-6 years	2001-2019	1.06*	Increase		2001-2019	1.02*	Increase
7-14 years	2001-2017	1.92*	Increase		2001-2019	1.35*	Increase
	2017-2019	-4.95	Decrease				
15-19 years	2001-2019	1.67*	Increase		2001-2019	1.51*	Increase
Eggs (g/d)							
1-6 years	2001-2019	-1.66*	Decrease		2001-2019	-1.58*	Decrease
7-14 years	2001-2019	-1.38*	Decrease		2001-2013	-1.88*	Decrease
					2013-2019	0.76	Stable
15-19 years	2001-2019	0.16	Stable		2001-2012	-0.32	Stable
					2012-2019	4.85	Increase
Milks (g/d)							
1-6 years	2001-2019	-0.90*	Stable		2001-2008	-3.05*	Decrease
					2008-2019	-0.04	Stable
7-14 years	2001-2019	-0.70*	Stable		2001-2019	-0.28	Stable
15-19 years	2001-2019	-2.21*	Decrease		2001-2019	-1.49*	Decrease
Fats and Oils (g/d)							
1-6 years	2001-2019	-1.05*	Decrease		2001-2019	-1.40*	Decrease
7-14 years	2001-2019	-0.82*	Stable		2001-2019	-0.65	Stable
15-19 years	2001-2019	-0.41	Stable		2001-2019	-0.59*	Stable
Confectioneries (g/d)							
1-6 years	2001-2017	-0.75	Stable		2001-2019	-1.43*	Decrease
	2017-2019	-25.82*	Decrease				
7-14 years	2001-2019	-0.50*	Stable		2001-2019	-0.80*	Stable
15-19 years	2001-2019	-1.73*	Decrease		2001-2019	-1.31*	Decrease
Beverages (g/d)							
1-6 years	2001-2019	2.13*	Increase		2001-2019	1.34	Increase
7-14 years	2001-2019	2.43*	Increase		2001-2019	2.51*	Increase
15-19 years	2001-2019	1.23	Increase		2001-2019	1.36*	Increase
Seasonings and Spices (g/d)							
1-6 years	2001-2016	1.23*	Increase		2001-2016	1.98*	Increase
	2016-2019	-14.61*	Decrease		2016-2019	-22.00*	Decrease
7-14 years	2001-2016	2.37*	Increase		2001-2007	5.89*	Increase
	2016-2019	-14.16*	Decrease		2007-2016	-0.22	Stable
					2016-2019	-10.99*	Decrease
15-19 years	2001-2003	8.68*	Increase		2001-2012	0.39	Stable
	2003-2016	-0.23	Stable		2012-2019	-5.81*	Decrease
	2016-2019	-10.83*	Decrease				
